# Getting to the Heart of Emotion Regulation in Youth: The Role of Interoceptive Sensitivity, Heart Rate Variability, and Parental Psychopathology

**DOI:** 10.1371/journal.pone.0164615

**Published:** 2016-10-14

**Authors:** Nele A. J. De Witte, Stefan Sütterlin, Caroline Braet, Sven C. Mueller

**Affiliations:** 1 Department of Experimental Clinical and Health Psychology, Ghent University, Ghent, Belgium; 2 Section of Psychology, Lillehammer University College, Lillehammer, Norway; 3 Department of Neurobiological Medicine, Oslo University Hospital – Rikshospitalet, Oslo, Norway; 4 Department of Developmental, Personality and Social psychology, Ghent University, Ghent, Belgium; Radboud Universiteit, NETHERLANDS

## Abstract

Emotion regulation and associated autonomic activation develop throughout childhood and adolescence under the influence of the family environment. Specifically, physiological indicators of autonomic nervous system activity such as interoceptive sensitivity and vagally mediated heart rate variability (HRV) can inform on emotion regulation. Although the effect of parental emotion socialization on emotion regulation appears to be influenced by autonomic processes, research on physiological regulation and the influence of parental factors remains scarce. This study investigated the relationship between self-reported habitual emotion regulation strategies and HRV at rest as well as interoceptive sensitivity in forty-six youngsters (27 female; age: *M* = 13.00, *SD* = 2.13). Secondly, the association between these autonomic correlates and parental psychopathology was also studied. Whereas better interoceptive sensitivity was related to reduced maladaptive emotion regulation, specifically rumination, high HRV was related to more use of external emotion regulation strategies (i.e., support seeking). In addition, increased HRV and decreased interoceptive sensitivity were associated with maternal internalizing and there was evidence for a possible mediation effect of HRV in the relationship between maternal internalizing and child external emotion regulation. This study elucidates the link between cognitive emotion regulation strategies and underlying physiological regulation in adolescents but also indicates a putative influence of maternal internalizing symptoms on emotion regulation in their offspring.

## Introduction

During late childhood and adolescence, emotion processing abilities are continuously developing in interaction with the family context [1]. Parents strongly shape emotional experience and emotion regulation (ER) in their children, but, interestingly, the effect of parental emotion socialization on ER appears to be partially mediated by autonomic processes [2]. How parental influences interact with a maturing autonomic nervous system is however still under study [3]. Previous studies already found indications that the development of emotional regulation during adolescence is associated with changes in autonomic activation [3, 4] and that such associations might be predictive of well-being and development of psychopathology including anxiety disorders and depression [5]. However, research on autonomic processes related to emotional regulation in youth remains limited and the precise role of parental factors in child autonomic nervous system activity and conscious awareness of this activity is insufficiently researched.

There is a long history of theories attributing importance to the role of the autonomic nervous system and bodily arousal in the experience of emotional states [6–10]. Bottom-up processes such as central nervous system processing of peripheral physiological reactions to emotional stimuli influence the subjective experience of emotional intensity [10]. However, individuals actively engage with the environment and apply regulatory control to an individually different extent, which makes both bottom-up and top-down processes key for emotional experience and regulation. Emotion regulation (ER) can be defined as “the extrinsic and intrinsic processes responsible for monitoring, evaluating, and modifying emotional reactions, especially their intensive and temporal features, to accomplish one’s goals” ([11]; pp 27). While the adaptive nature of a specific strategy also depends on situational factors and environmental demands [12], the large spectrum of ER strategies can generally be categorized into adaptive, maladaptive, and external ER. A well-known example of an adaptive strategy is cognitive reappraisal, the cognitive reinterpretation of situations to change the emotional response to them [13]. Rumination on the other hand is an example of a maladaptive strategy and is characterized by a repetitive and passive focus on symptoms of distress and possible causes and consequences [14]. Finally, interpersonal strategies such as support seeking [3] are termed external ER strategies. There is currently no consensus on whether external strategies are beneficial or detrimental to wellbeing [15]. A meta-analytic review showed that although ER is far less studied in youth than adulthood and youngsters might be less able to use more demanding strategies such as problem solving, children do already use both adaptive and maladaptive strategies to manage their emotions [16]. However, the psychophysiological correlates of these ER strategies in youth have not yet been fully characterized.

Top-down regulation of emotional states has often been linked to a specific autonomic component, i.e. vagally mediated intrinsic variability in heart rate, by both theory and research. Vagally mediated heart rate variability (HRV) refers to the beat-to-beat variability in heart rate and is controlled by the parasympathetic nervous system through the vagus nerve. It is an index of healthy functioning and flexibility to adapt to complex environmental demands [17]. The neurovisceral integration model states that vagally mediated and prefrontally modulated cortico-cardiac interaction at resting state can be considered a proxy for prefrontal inhibitory capacity and hence for ER capacity [17, 18]. In adults, higher vagally mediated resting HRV corresponds to higher ability to react and return to homeostasis (i.e. regulate) [19] and is related to lower levels of affective instability [20], greater (increase in) behavioral regulatory control [21, 22], improved cognitive control over unwanted memories [23], better fear extinction [24], and less difficulties in self-reported ER [25]. By contrast, research in children is more limited and to date inconsistent. While Vasilev et al. [5] showed that developmental changes in resting HRV were related to self-reported ER abilities in children and two other studies showed that higher resting HRV is associated with better effortful control and increased perceived control over anxiety [26, 27], Gentzler et al. [28] did not find an association between baseline HRV and adaptive or maladaptive ER in youth. Interestingly, a second theory, the polyvagal perspective, supports the supposition that vagally mediated HRV is related to regulation, but additionally proposes that it is associated with social behavior. The polyvagal theory states that the vagus nerve is involved in the inhibition of primitive neural fight and flight mechanisms and the promotion of social behavior [9, 29]. Therefore, increased vagus control is associated with higher HRV as well as emotion expression, social competence and active engagement with the environment [30, 31]. Again, some studies in adults have already supported the relationship between HRV and social support seeking [29, 32], however no studies on the association between resting HRV and external ER in youngsters could be found. In conclusion, while HRV has been reliably shown to be associated with (interpersonal) self-regulation by both theory and empirical research in adults, this association requires further validation in adolescents.

As opposed to top-down emotional control processes, the psychophysiological correlates of bottom-up processes are often disregarded in the ER literature. However, these bottom-up emotional processes have also been linked to a specific physiological process, i.e. interoceptive sensitivity (IS), by the somatic marker hypothesis [10]. The somatic marker hypothesis states that the subjective emotional experience is shaped by bodily conditions [33]. Sensitivity towards these bodily conditions and more specifically, towards changes in one’s physiological state as represented by IS to one’s heartbeat, has been shown to moderate the effect of bodily responses on emotional arousal [34, 35]. This is further related to individual differences in subjectively experienced emotional intensity [36, 37]. Research in adults indicates that high IS is linked to greater emotional arousal [38, 39], less alexithymia [40], and, interestingly, also better downregulation of negative affect via cognitive reappraisal [41]. Research on the associations between IS and emotional states in children is limited. One available study in primary school children showed that IS was significantly associated with interpersonal emotional intelligence and adaptability (i.e. ability for behavioral adjustment in a changing environment; [42]). Although initially IS was conceptualized as being involved in bottom-up emotion experience, other available evidence links it to regulation as well [41]. Higher IS could be related to more precise bodily feedback, i.e., better information and central representation of the visceral states caused by emotions, and therefore regulation might be applied more effectively. While no studies investigated the relationship between parental factors and child IS, the biological sensitivity to context theory suggests that individual differences in child psychophysiology emerge from, and constantly interact with, the family environment to predict psychiatric and biomedical outcomes [12, 43].

The family environment might not only create a context which puts children at increased risk for maladaptive outcomes [44], but parental factors might also interact with psychophysiological processes such as HRV in the offspring. While high vagal control is generally considered to be related to positive outcomes such as ER capacity and regulatory control [17, 18], it has also been reported to exacerbate the negative effect of maternal depressive symptoms on child ER [45]. Furthermore, direct effects of parental psychopathology suggest that offspring of patients with an anxiety disorder or depression have decreased cardiac vagal function [46, 47]. However, these studies all recruited participants in early to mid-childhood and age has proven to be an important determinant of HRV. Evidence suggests that individuals may be more likely to show HRV differences in certain developmental periods critical for the augmentation of the autonomic nervous system or self-regulatory skills [48]. In early childhood, at-risk children with a parental history of mood disorders and low-risk peers show a similar level of HRV functioning. By comparison, in late childhood and adolescence, sensitivity to environmental influences of stress and challenges to self-regulatory skills increase and this is reflected in higher inter-individual variability in HRV between high-risk and low-risk children [48]. The relationship between individual differences in parents and adolescent psychophysiology and ER is complex and insufficiently addressed in previous research. Furthermore, the precise roles of HRV and IS in ER remain unclear.

Therefore, this study had three goals. First, we aimed to examine the link between HRV and IS to ER strategies (adaptive, maladaptive, external) in late childhood and adolescence. Second, to investigate the influence of parental factors on physiological reactivity in their offspring in late childhood and adolescence. Third, to explore whether autonomic responses could mediate the relationship between parental internalizing (i.e., anxiety and depressive) symptoms and child ER. Based on recent evidence, we anticipated that better resting HRV (e.g., [21]) and IS [41] would be associated with better regulatory control and adaptive ER and, in case of HRV, also with increased use of external ER (e.g., [29]). Second, limited previous evidence suggests that anxiety disorders in the parents are correlated with HRV in the child [46]. Based on this prior study, one would expect that increased parental internalizing psychopathology is associated with decreased HRV in the child. Furthermore, we would like to investigate the relationship between parental internalizing psychopathology and child IS.

## Materials and Methods

### Participants

Forty-six healthy youngsters ages 9 to 16 years (22 female; Age: *M* = 13.00, *SD* = 2.13) participated in this study. Initially, 50 youngsters (mostly of Caucasian ethnicity from a middle-class background) were recruited from the extended urban area of Ghent (Belgium) by advertisement in local schools and youth organizations. These participants were invited for participation by telephone and all participants as well as their parents filled out informed consent forms (parents) and assent forms (youths). All participants received a fee for expense reimbursement (€ 20). Ethical approval was obtained from the ethical committee of Ghent University Hospital. Exclusion criteria consisted of an internalizing DSM-5 disorder or attention deficit hyperactivity disorder (ADHD), and severe medical or neurological illnesses. Four participants had to be excluded due to the presence of a DSM-5 disorder (n = 3) or a neurological illness (n = 1). To detect the presence of DSM disorders the Dutch Structured Clinical Interview for DSM-IV, Child Edition (KID-SCID [49, 50]) was conducted by a psychologist or psychology student trained in conducting the KID-SCID. Since this interview was constructed to assess DSM-IV criteria, several questions were added to ensure coverage of all DSM-5 criteria. None of the mothers were confronted with current or past psychiatric disorders, one father suffered from bipolar disorder.

### Psychophysiological measures

#### Heart rate and resting heart rate variability

The ECG was collected at a sampling rate of 1000 Hz with de ECG100C module of the Biopac MP150 acquisition system and electrodes (small stress test electrode EL501) were positioned in a modified Einthoven lead II configuration (Biopac systems, Goleta, CA, United States of America). One electrode was placed just below the right clavicle, the second on the left lower torso, and the ground electrode was placed on the right lower torso. Heart rate was recorded during 8 consecutive minutes while watching a segment of an animated movie (Wall-*E*) that contained very few emotionally salient events or speech. To increase reliability (related to settling into the task, or activity of the experimenter) of these 8 minutes, the first 150 and last 30 seconds were excluded to get a reliable measure of 5 consecutive minutes. In case there were large artifacts (in the form of irregularities or movement artifacts that could not be corrected by hand) in the middle 5 minutes, the entire 8 minutes were inspected and the 5 minutes of the highest quality (i.e. the smallest amount of measurement artifacts) were selected. Inter beat intervals and HRV were calculated using ARTiiFACT software [51]. We analyzed the two most frequently used HRV indices of the time- and frequency domain, respectively the root mean square of successive differences (RMSSD) and the high frequency component (HF; Fast-Fourier-Transformation, bandwidth 0.15 to 0.4 Hz) [52].

#### Interoceptive sensitivity

Previous research has already shown that the heartbeat detection task (used to assess IS) can capture individual differences in children even though children do experience more difficulty in executing and/or feeling their heartbeat, as shown by lower mean IS scores relative to adults [42]. In this task, participants were instructed to count their heartbeats (without taking their pulse) in the interval between two auditory signals. To account for (and rule out) counting ability as a potential confounding factor in our young sample, participants were instructed to count the number of heartbeats in an auditory sample of a heart beating (not their own) before doing the actual IS task. In addition, to make the task more engaging, participants could listen to their own heartbeat through a stethoscope before commencing the task. Participants completed one practice trial of 20 seconds followed by 6 experimental trials of 25, 35, and 45 seconds (precise order: 25, 35, 45, 35, 45, 25). Each trial was followed by two questions: ‘How many heartbeats did you count?’ and ‘How sure are you on a scale of 1 (not sure at all) to 9 (very sure)?’. The second question explores the metacognitive belief in ones interoceptive abilities (also referred to as interoceptive awareness [53]). The number of actual heartbeats in each trial was recorded with ECG (see section 2.2.1.). The IS score was calculated as follows, with a higher score indicating better performance [42]:

16∑[1−(|actual heartbeats−counted heartbeats|actual heartbeats)]

### Questionnaires

#### Emotion regulation

To assess ER strategies the FEEL-KJ was used [15, 54]. The validity of this questionnaire has already been proven by previous studies [55, 56]. The Dutch version of the FEEL-KJ contains 90 items that assess to what extent children and adolescents habitually use adaptive, maladaptive, and external ER strategies [15]. The scale of adaptive strategies contains the subscales problem-oriented action, cognitive problem-solving, acceptance, forgetting, distraction, reevaluation, and evoking positive mood. Maladaptive strategies are represented by giving up, aggression, withdrawal, self-devaluation, and rumination. Finally, external ER consists of interpersonal strategies to regulate emotions and is represented by the subscales social support seeking, expression, and emotional control. While the subscale expression refers to openly displaying how you feel, emotional control concerns to what extent emotions are being concealed from others and is consequently reverse scored. Each ER strategy is represented by two items that are repeated in three different emotion categories (angry, scared, and sad) and participants are asked to rate the frequency of use of each item on a 5-point scale. The FEEL-KJ questionnaire has a satisfactory overall validity and reliability (Cronbach’s alpha of 0.86 for the entire questionnaire in this sample).

#### Regular exercise and pubertal development

Since both HRV and IS could be influenced by the level of physical fitness [52, 57], participants were asked to report whether they regularly exercise (yes or no) and list sportive activities. In addition, previous research has shown that pubertal development has an impact on cardiac contractility [58] and HRV [59, 60]. Furthermore, the influence of pubertal development on HRV should be considered, certainly in the context of parental psychopathology [48]. Therefore, a self-rated version (consisting of 9 items for girls and 8 items for boys) of the pubertal development scale (PDS [61]) was used. The reliability of this scale was adequate (Cronbach’s alpha in this sample of .83 and .75, for boys and girls, respectively) and previous research had already shown that the PDS is a good scale for the assessment of physical maturity [62].

#### Parental psychopathology

A Dutch translation of the 14-item Hospital Anxiety and Depression Scale (HADS; [63, 64]) was used to measure symptoms of anxiety and depression in the parents. Although the Dutch version of this scale has been validated in previous research, a better balance between sensitivity and positive predictive value has been found in the total HADS score than in the two factor solution (based on 7 items each [64]). Therefore, a total internalizing sum score was calculated (Cronbach’s alpha of .84 in this sample).

### Procedure

Before testing, children and adolescents completed the FEEL-KJ and filled out the two questions regarding physical exercise and sports. Similarly, both parents were asked to fill out the HADS and the questions regarding psychiatric disorders before coming to the laboratory. All questionnaires were completed on a secure online platform hosted by the Department of Developmental, Personality and Social Psychology of Ghent University. On the day of testing, participants started with participating in the DSM-5 adapted KID-SCID interview. Subsequently, they were weighed and measured and prepared for the physiological recordings. After recording ECG at rest, the PDS was completed. Subsequently, participants were asked whether they had ever heard their heartbeat and given the chance to listen to their heart with a stethoscope, right before they executed the heart beat detection task. The study protocol also included an ER training and an experimental Physiological Indicators of Emotion Regulation (PIER) task that were collected after the present data. However, given that the training and task investigated a different research question, they will be reported elsewhere.

### Data analysis

#### Primary analysis: correlations of emotion regulation abilities with psychophysiology

To investigate the relationship between the physiological correlates (HRV and IS) and ER as well as parental psychopathology, partial correlation tests were calculated (alpha = .05, two-tailed) with the SPSS software package (version 20, IBM, Chicago, IL, USA). Both HRV measures violated the assumption of normality and were log-transformed (log10) to achieve normal distribution. These log-transformed scores were used in all further analyses. There were some missing values in IS score (n = 2, 2 female), PDS score (n = 1, male), regular exercise score (n = 1, female) and HADS internalizing in the mother (n = 1, male) and father (n = 4, 2 female) due to equipment failure, non-compliance, or unavailability of parents. Data was missing completely at random and therefore we imputed the missing values through expectation maximization (by use of the SPSS missing values analysis module) to provide unbiased parameter estimates and improve the statistical power of the analyses. In accordance with previous research, gender could confound results for both IS (*r*(46) = .33, *p* = .02; higher IS in males vs. females) and HRV (RMSSD: *r*(46) = .27, *p* = .07; higher HRV in males vs. females) [42, 65], regular exercise could additionally distort HRV outcomes (RMSSD: *r*(45) = .30, *p* = .05; higher HRV in high vs. low regular exercise) [66], and body mass index (BMI) is a possible confound for IS (*r*(46) = -.29, *p* = .05; higher IS in low vs. high BMI) [67]. Consequently, these factors were included as potential confounders in the partial correlations. Furthermore, since there was a large age range in the sample and consequently varying levels of pubertal development, all following analyses were additionally controlled for PDS score.

#### Exploratory analysis: mediation model

Although mediation effects were not initially hypothesized, the primary data analyses gave rise to some interesting research questions that deserved further investigation through mediation analyses. Therefore, to further interrogate our data and investigate how parental internalizing could have indirectly influenced child ER, exploratory mediation models were performed using the PROCESS 2.15 macro [68] in SPSS. For significance testing the bootstrap method (with 5000 samples) was preferred over the Sobel test since the latter is only suited for use in large samples and has lower power [69–71]. Similar to the partial correlations, gender and PDS score were always entered as covariate in the mediation analyses, while regular exercise was added only in the case of HRV and BMI was only added for IS.

## Results

### Interoceptive sensitivity, resting HRV, and emotion regulation

Table 1 provides an overview of the demographics of the current sample. RMSSD (log10) and HF (log10) were significantly positively associated with external ER but not adaptive or maladaptive ER (Table 2). This correlation indicated that higher resting HRV (both RMSSD and HF) was associated with increased use of external ER strategies. By comparison, IS was significantly negatively correlated with maladaptive ER but not adaptive or external ER (Table 2). Better IS was associated with less use of maladaptive ER. Interestingly, the participant’s belief about interoceptive abilities was not correlated with IS nor adaptive, maladaptive, or external ER. Of note, IS and resting HRV were not correlated.

**Table 1:** Study sample characteristics (*n* = 46).

	Mean (*SD*)	Range (min-max)
**Children**		
Age	13.00 (2.13)	9–16
Ratio Female/male	27/19	
Interoceptive sensitivity	.56 (.20)	.08–1.00
Interoceptive certainty	5.25 (1.81)	1.33–9.00
Heart rate variability		
• RMSSD	61.38 (33.84)	9.61–188.08
• RMSSD (log10)	1.73 (.23)	0.98–2.27
• High frequency	1925.58 (2388.10)	36.07–12580.84
• High frequency (log10)	3.04 (.50)	1.56–4.10
Pubertal development score	1.64 (.77)	0.33–3.33
Habitual emotion regulation (FEEL-KJ)		
• Adaptive emotion regulation	137.15 (24.08)	76–181
• Maladaptive emotion regulation	75.43 (15.32)	37–113
• External emotion regulation	55.28 (11.21)	34–80
**Parental psychopathology**		
Internalizing symptoms (HADS)		
• Mother	8.76 (5.71)	0–22
• Father	7.40 (5.04)	0–25

**Table 2:** Partial correlations between interoceptive sensitivity and heart rate variability and ER strategies and parental internalizing.

	Interoceptive sensitivity (*n* = 46)	Heart rate variability (*n* = 45)	
		RMSSD (log10)	HF (log10)
Adaptive ER	-.25	.06	.06
Maladaptive ER	-.31*	.11	.06
External ER	-.10	.31*	.31*
Maternal internalizing	-.42**	.39**	.37*
Paternal internalizing	-.13	-.21	-.13

Significant correlations are presented in bold font. All correlations were controlled for gender and pubertal development, interoceptive sensitivity was additionally controlled for BMI and heart rate variability was additionally controlled for regular exercise.

*p < .05,

**p < .01.

### Subtypes of emotion regulation

To assess from which specific subscales of ER strategies the significant correlations emerged, we conducted further analyses. These analyses indicated that the correlation between high HRV and external ER was mainly driven by high support seeking (Table 3). When looking at the correlation between maladaptive ER and IS, this correlation was driven by rumination and self-devaluation (Table 4), where higher IS was associated with decreased rumination and self-devaluation.

**Table 3:** Follow-up correlations between heart rate variability and the subscales of external emotion regulation.

	Heart rate variability	
	RMSSD (log10)	HF (log10)
Support seeking	.39*	.37*
Expression	.23	.21
Emotional control	.04	.06

All correlations were controlled for gender, puberty, and regular exercise. Significant correlations are presented in bold font. *n* = 45;

*p < .05;

**Table 4:** Follow-up correlations between interoceptive sensitivity and the subscales of maladaptive emotion regulation.

	Interoceptive sensitivity
Giving up	-.18
Aggression	-.15
Withdrawal	-.05
Self-devaluation	-.31*
Rumination	-.35*

All correlations were corrected for puberty, BMI, and gender. Significant correlations are presented in bold font. N = 46;

*p < .05;

### Parental psychopathology and child physiology

**Fig 1:**
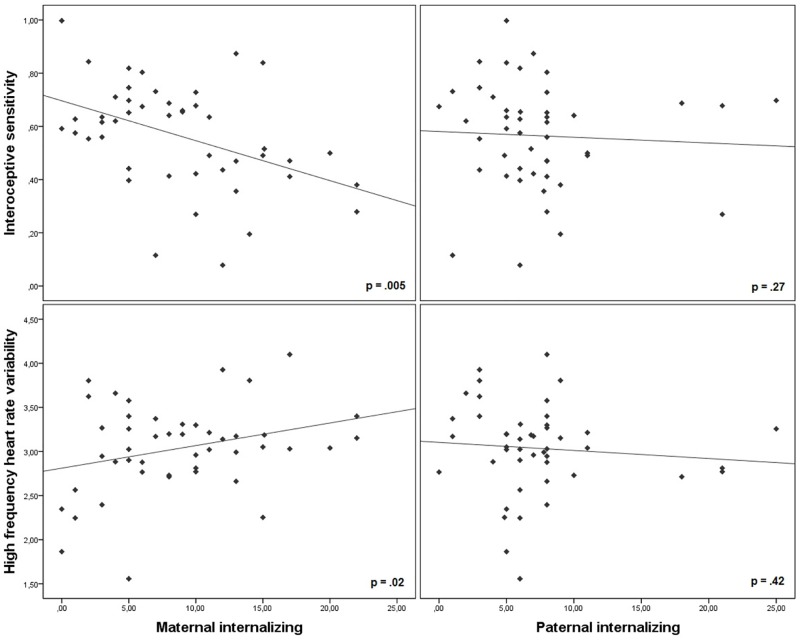
Scatterplots depicting the significant correlations between parental internalizing symptoms and interoceptive sensitivity and heart rate variability (high frequency component) in the child. While maternal internalizing symptoms were correlated with child autonomic components (left panes), this effect was absent for paternal internalizing symptoms (right panes). Bivariate correlations are shown for the purpose of clear visualization and interpretation.

Correlations between internalizing scores of parents and physiological indices of the child revealed that maternal internalizing symptoms were significantly related to child RMSSD, HF, and IS, while paternal internalizing symptoms were not (Table 2). Specifically, high internalizing in the mother was associated with higher HRV and lower IS (Table 2, Fig 1). Maternal internalizing was significantly directly negatively correlated with adaptive ER (*r*_partial_(43) = -.31, *p* = .04) and positively with maladaptive ER (*r*_partial_(43) = .30, *p* < .05) in the child, but not external ER (*r*_partial_(43) = -.04, *p* = .81). These correlations indicate that anxiety and depressive symptoms in the mother were associated with lower habitual use of adaptive and higher use of maladaptive ER strategies in the offspring. There were no significant correlations between paternal internalizing symptoms and adaptive (*r*_partial_(43) = .002, *p* = 1.00), maladaptive (*r*_partial_(43) = .26, *p* = .08), or external (*r*_partial_(43) = -.07, *p* = .63) ER in the child.

### Exploratory mediation analyses

**Fig 2:**
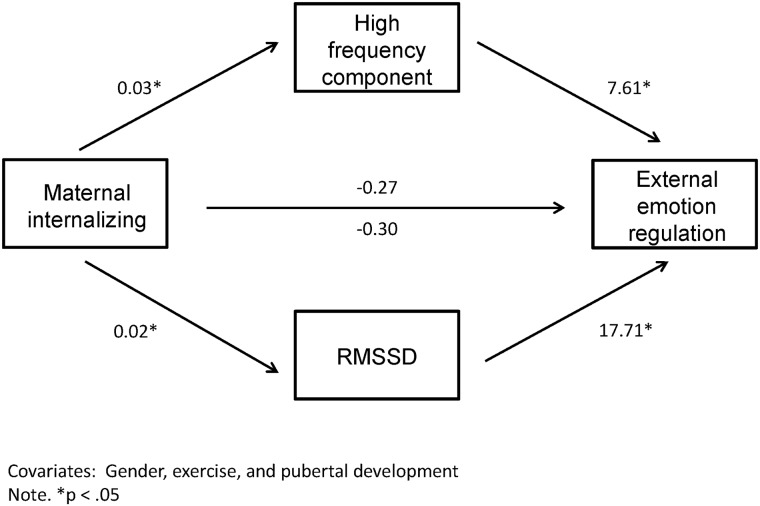
Visual representation of the significant indirect effect of maternal internalizing on external emotion regulation in the child through both measures of heart rate variability (the high frequency component and RMSSD).

Based on the correlations mentioned above, there are three mediation models that deserve investigation. Firstly, maternal internalizing could impact HRV which in turn could influence external ER (Fig 2). There was evidence for an indirect effect of maternal internalizing on external ER through high frequency HRV (log10) (*b* = 0.25, 95% bootstrap CI: 0.01–0.57). The completely standardized indirect effect was 0.14 (95% bootstrap CI: 0.01–0.31). There was also an indirect effect of maternal internalizing measure on external ER through RMSSD (log10) (*b* = 0.27, 95% bootstrap CI: 0.03–0.58. The completely standardized indirect effect was 0.15 (95% bootstrap CI: 0.01–0.31). Finally, maternal internalizing could have an impact on maladaptive ER through IS. However, mediation analysis showed that there was no indirect effect (*b* = 0.23, 95% bootstrap CI: -0.03–0.71). The completely standardized indirect effect was 0.10 (95% bootstrap CI: -0.01–0.30).

## Discussion

Because research on autonomic processes related to emotional processing in youth is still in its infancy, the present study had three main goals. First, it aimed to examine the relationship between autonomic correlates (HRV at rest and IS) and habitual ER use in children and adolescents. Second, it investigated the relationship between parental internalizing psychopathology and the autonomic correlates of ER in their offspring. Finally, exploratory mediation analysis was performed to study possible indirect effects. With regards to the first goal, higher HRV was associated with increased use of external ER, and support seeking in particular. By contrast, higher IS was related to decreased use of maladaptive ER, specifically rumination and self-devaluation. As for the second goal, maternal but not paternal internalizing symptoms were related to increased HRV and decreased IS. Thirdly, we did find evidence for a possible mediation effect of HRV in the relationship between maternal internalizing and child external ER. These results highlight the importance of autonomic processes in the context of ER in youth.

According to the polyvagal theory, high vagal control of autonomic nervous system activity is associated with emotion experience, top-down regulation, and social behavior [9]. Consistent with this theory, HRV was positively associated with external ER, such as support seeking. This finding is also consistent with work in adults that supports the association between vagally mediated HRV at rest and prosocial behavior such as engagement coping and social wellbeing [29]. The present findings are consistent with this perspective and indicate that higher resting HRV in youth is associated with increased habitual use of strategies that require a social context, i.e., external ER. Given the continuous development of ER in adolescents, interpersonal regulation might not only aid in momentary emotion experience, but also allow adolescents to learn from the environment and develop a broader repertoire of ER skills for future purposes. Nevertheless, HRV did not correlate with adaptive ER, a finding which is in line with Gentzler et al. [28] but contrary to what other previous research has suggested [5, 21]. A possible cause of this inconsistency in the literature lies in the different interpretations of the role of resting HRV in ER. While in some studies HRV was related to adaptive ER specifically (e.g., [21]), others suggested that resting HRV is involved in the flexibility of using different ER strategies [72], while yet others propose that high resting HRV could facilitate the acquisition of different adaptive ER strategies in children [45]. While the perspective of Blandon et al. [45] is most in line with our findings regarding prosocial behavior, we cannot exclude nor confirm these perspectives since the FEEL-KJ only assesses habitual ER strategy use and not flexibility in ER use, acquisition of ER, or ER efficacy. A second possible explanation for the discrepancy between current and previous research can be found in previous limitations to focus on a limited selection of ER strategies/skills, whereas the current study registered routine use of a wider range of different ER strategies in daily life.

Similarly to HRV, IS also correlated with a specific category of ER strategies. Increased IS was significantly associated with decreased use of maladaptive ER strategies, particularly rumination. This association seems to be independent from the metacognitive belief of one’s interoceptive skills, which did not correlate with IS nor ER. Such a result is consistent with previous literature considering interoceptive sensitivity and awareness to be two dissociable constructs [73, 74]. Currently, the link between IS and (maladaptive) ER has rarely been investigated, however, there is evidence supporting specifically low IS as a vulnerability factor for adult psychopathology known to be characterized by maladaptive ER [16], such as depression [75], personality disorders [76], and possibly anxiety disorders [77]. The present association of better IS with lower use of self-devaluation and rumination suggests that increased accuracy for emotional states might play a role in the prevention of a repetitive negative thinking style, at least in healthy adolescents. However, given the scarcity of available literature, the relationship between IS and maladaptive ER requires further examination preferably in both clinical and non-clinical groups across various ages. Taken together, both higher IS and HRV contribute to healthy ER in a different way. Better bottom-up emotional awareness, in the form of IS, is associated with decreased use of maladaptive ER, while high top-down vagally mediated HRV contributes to interpersonal regulation.

The second aim concerned identifying the role of parental psychopathology in psychophysiological correlates of ER. While some evidence supports the importance of parental (mainly maternal) factors in child ER [2, 78], research on the effects of maternal and paternal psychopathology on the physiological components of ER in adolescence is rare. In the present study, we found an association between maternal, but not paternal, internalizing symptoms and child psychophysiology. Specifically, high maternal internalizing problems in healthy mothers were associated with higher resting HRV in youngsters. Although this finding is opposite to Srinivasan et al. [46], who reported decreased cardiac vagal function in children of mothers and fathers with panic disorder, a possible explanation for this discrepancy lies in the sample used. The current study, as opposed to the study by Srinivasan and colleagues [46], did not select parents based on prior psychiatric diagnosis. In fact, no mothers in the present study reported current or past psychiatric disorders. This suggests that protective factors, such as high HRV [79] or increased interpersonal regulation [80], could reduce disease burden in these mothers. These protective factors could in turn be passed on to the children. This hypothesis is supported by the additional, exploratory mediation analyses. These analyses indeed suggested that the effect of maternal internalizing on external ER could be mediated by HRV measures. In conclusion, healthy mothers with internalizing symptoms appear to have children with higher HRV, which in turn fosters strategies that promote adaptive functioning in the children.

In contrast to HRV, in this study, maternal internalizing symptoms were associated with lower IS in children. Previous studies have already shown decreased IS to be related to internalizing disorders in adults [75, 77]. Perhaps these mothers with internalizing symptoms might have compromised IS themselves and consequently transmit this vulnerability factor to their children, hereby increasing their offspring’s risk for developing maladaptive ER (and possibly psychopathology). However, the additional, exploratory mediation analysis does not support an indirect effect of maternal internalizing on maladaptive ER through IS. Similarly to HRV, paternal internalizing symptoms do not influence IS. We could speculate that since mothers are often the primary caregiver in the family, they could have a larger impact on child behavior. However, future research is needed to investigate which factors underlie this gender difference. Furthermore, while the means by which decreased IS skills are conveyed from mother to child remain unknown, it is known that HRV is influenced both by genetic and environmental factors [81, 82]. We speculate that next to genetic transmission problematic parent-child interactions and heightened family stress associated with parental psychopathology could result in general dysregulation and related psychophysiological response patterns in children [82]. However, this study does not allow us to specify to what extent parental influences are transmitted through genetics or such environmental processes. Further research investigating possible mediation effects of parenting factors or genetics in the relationship between parental psychopathology and IS and HRV is needed. In conclusion, mothers with internalizing symptoms appear to have children with low interoceptive skills and these children appear to make more use of maladaptive ER strategies. However, at the same time these mothers provide their children with (potentially) compensatory tools (i.e. HRV) that promote ER and healthy interactions with the environment. However, mediation analyses in larger samples need to confirm these results.

Some limitations require discussion. Contrary to resting HRV protocols in adults, in which participants are asked to sit still, relax, and focus on fixation point, a movie clip was shown during resting HRV to elicit stronger task compliance and avoid potential confounds related to movement in the youngsters. Although we cannot exclude that some emotional response was evoked, the chosen clip was low in emotional arousal and included very few speech components. Moreover, previous authors have also opted for using neutral movie clips to assess baseline reactivity in children (e.g., [45, 83–85]). Likewise, to increase engagement and promote compliance for the IS task, children had the opportunity to listen to their heart with a stethoscope immediately prior to the task. However, this could increase insight into their heart rate and hereby improve the accuracy of the estimated number of heartbeats during the task. Future research could benefit from increasing the time between listening to the heart and actually performing the task to diminish the chance of this affecting task performance. Whereas prior work has indicated that regular exercise could impact IS [57] and BMI could influence HRV [86], the absence of correlations of these variables with IS and HRV indicates little influence in the present data. The current study is novel in investigating the associations between two complementary psychophysiological indices and ER and parental psychopathology in adolescence but it also raises some interesting questions for future research. Contrary to previous research [27, 28], the current study suggested that gender might influence the effects. Furthermore, this is a cross-sectional study in Caucasian middle class youngsters. Therefore, future research should explore the effects of gender and different ethnic and socio-economic backgrounds throughout development to increase generalizability. Additionally, given that ER is associated with psychopathology [87], it would be interesting to reproduce the current study in adolescents with psychiatric disorders.

In conclusion, the current study provided evidence on the complementary nature of two processes involved in physiological regulation and ER in adolescents. While high IS was associated with low maladaptive ER, high HRV was associated with external ER. Moreover, maternal internalizing symptoms were associated with both physiological indices in their children, specifically with higher HRV but lower IS, suggesting an interesting relationship between maternal psychological problems and autonomic processes related ER (as a potential protective factor) in their children. Future work will need to replicate these findings in psychiatric samples and examine how these factors could provide risk or resilience factors longitudinally.
